# Extensive Pericentric Rearrangements in the Bread Wheat (*Triticum aestivum* L.) Genotype “Chinese Spring” Revealed from Chromosome Shotgun Sequence Data

**DOI:** 10.1093/gbe/evu237

**Published:** 2014-10-27

**Authors:** Jian Ma, Jiri Stiller, Yuming Wei, You-Liang Zheng, Katrien M. Devos, Jaroslav Doležel, Chunji Liu

**Affiliations:** ^1^CSIRO Agriculture Flagship, St Lucia, Queensland, Australia; ^2^Triticeae Research Institute, Sichuan Agricultural University, Wenjiang, Chengdu, China; ^3^Department of Crop & Soil Sciences, and Department of Plant Biology, University of Georgia; ^4^Centre of the Region Haná for Biotechnological and Agricultural Research, Institute of Experimental Botany, Šlechtitelů, Olomouc, Czech Republic; ^5^School of Plant Biology, The University of Western Australia, Perth, Western Australia, Australia

**Keywords:** chromosomal rearrangement, comparative genomics, pericentric inversion, pericentromeric regions, translocation, Chinese Spring

## Abstract

The bread wheat (*Triticum aestivum* L.) genotype “Chinese Spring” (“CS”) is the reference base in wheat genetics and genomics. Pericentric rearrangements in this genotype were systematically assessed by analyzing homoeoloci for a set of nonredundant genes from *Brachypodium distachyon*, *Triticum urartu*, and *Aegilops tauschii* in the CS chromosome shotgun sequence obtained from individual chromosome arms flow-sorted from CS aneuploid lines. Based on patterns of their homoeologous arm locations, 551 genes indicated the presence of pericentric inversions in at least 10 of the 21 chromosomes. Available data from deletion bin-mapped expressed sequence tags and genetic mapping in wheat indicated that all inversions had breakpoints in the low-recombinant gene-poor pericentromeric regions. The large number of putative intrachromosomal rearrangements suggests the presence of extensive structural differences among the three subgenomes, at least some of which likely occurred during the production of the aneuploid lines of this hexaploid wheat genotype. These differences could have significant implications in wheat genome research where comparative approaches are used such as in ordering and orientating sequence contigs and in gene cloning.

## Introduction

The bread wheat (*Triticum aestivum* L.) genotype “Chinese Spring” (CS) has been used worldwide as the reference base in wheat research. Many valuable genetic stocks have been developed in the CS background. They include complete series of nullisomic-tetrasomic lines (Sears 1954; Sears et al. 1966), telocentric lines (Sears ER and Sears L 1978), and other deletion stocks (Endo and Gill 1996). Not surprisingly, this genotype is also the one selected for sequencing by the International Wheat Community (Gill et al. 2004; Paux et al. 2008; Brenchley et al. 2012; International Wheat Genome Sequencing Consortium [IWGSC] 2014).

Due to the highly conserved gene order among different grass genomes (Moore et al. 1995; Devos 2005; Vogel et al. 2010), comparative information has been widely used to order and orientate contigs in wheat genome sequence assemblies (Berkman et al. 2011, 2012; Brenchley et al. 2012; Hernandez et al. 2012). However, previous studies have shown some structural differences among chromosomes of the three bread wheat subgenomes. In addition to the well-known cyclic translocations involving chromosomes 4A, 5A, and 7B (Liu et al. 1992; Devos et al. 1995; Ma et al. 2013), there is also strong evidence for the presence of a number of pericentric inversions. Two of them, one in chromosome 2B and the other in 5A, were detected by deletion-bin mapping of ESTs (expressed sequence tags) (Conley et al. 2004; Linkiewicz et al. 2004; Miftahudin et al. 2004). Restriction Fragment Length Polymorphism (RFLP)-based mapping of ESTs to individual chromosome arms using wheat aneuploid stocks and wheat-alien ditelosomic addition lines provided evidence for the existence of pericentric inversions in chromosomes 3B and 6B (Qi et al. 2006).

Previous analyses of chromosomal rearrangements based on cytology and marker gene locations had limitations. The various techniques of cytology may only be able to detect rearrangements involving large chromosomal segments. For example, induced chromosome pairing is generally limited to detecting homoeologous sequences at the termini of chromosomes (Naranjo et al. 1987). Although a recent publication reported the successful application of single gene FISH (fluorescent in situ hybridization) in wheat (Danilova et al. 2014) which could significantly enhance the power of cytology, the best resolution of FISH used in previous studies was about 70 kb on stretched chromosomes (Valárik et al. 2004).

The resolution based on marker gene locations is high but the numbers of markers used in previous studies were limited. Some of the putative rearrangements were based on locations of a single or a very small number of markers (Blanco et al. 1998; Conley et al. 2004; Qi et al. 2006). As gene losses (Song et al. 1995; Feldman et al. 1997; Ozkan et al. 2001) and duplications (Salse et al. 2008; Brenchley et al. 2012) are common in polyploid wheat, many genes detect multiple loci belonging to both homoeologous as well as nonhomoeologous chromosomes. As a result, some of the putative rearrangements deduced from the locations of one or a small number of genes can be unreliable (Ma et al. 2013).

Taking advantage of the recent progress in genome sequencing, the chromosome arm locations of a set of nonredundant genes identified from *Brachypodium distachyon, T. urartu,* and *Aegilops tauschii* were determined based on the shotgun sequence obtained from individual chromosome arms of the bread wheat genotype CS. We used the data to systematically analyze the presence of rearrangements differentiating the three homoeologous genomes of CS.

## Materials and Methods

### Data Collection

Gene-coding sequences (CDS) from *Brachypodium* genome version 1.2 were downloaded from http://www.plantgdb.org/BdGDB (last accessed November 3, 2014) (Vogel et al. 2010). Wheat deletion bin-mapped ESTs for individual deletion bins were downloaded from GrainGenes 2.0 (http://wheat.pw.usda.gov/GG2/index.shtml, last accessed November 3, 2014) (Carollo et al. 2005). CDS of *A. tauschii* (wheatD_final_43150.gff.cds) (Jia et al. 2013) and *T. urartu* (TRIUR3_120813_filter150_cds) (Ling et al. 2013) were downloaded from GIGA_DB (http://gigadb.org/, last accessed November 3, 2014). CS chromosome arm shotgun sequences were from the IWGSC (http://www.wheatgenome.org/, last accessed November 3, 2014) (IWGSC 2014). The pseudomolecule sequence of CS chromosome 3B (traes3bPseudomoleculeV1) was downloaded from https://urgi.versailles.inra.fr/gb2/gbrowse/wheat_annot_3B/ (last accessed November 3, 2014) hosted by Unité de Recherche Génomique Info (Choulet et al. 2014).

### Analysis of Pericentric Rearrangements

Reciprocal BLASTn searches were used to identify a set of nonredundant orthologous gene sequences among deletion bin-mapped wheat ESTs and CDSs of *Brachypodium*, *T. urartu,* and *A. tauschii*. This was done using the *Brachypodium* genes as the basis. Genes of *T. urartu* without an ortholog in the *Brachypodium* gene set were then added to the list of the unique orthologous sequences, which was followed by genes from *A. tauschii* and deletion bin-mapped wheat ESTs*.* The nonredundant orthologous sequences assembled from these different sources were then analyzed against the CS shotgun sequences using the BLAST + BLASTn algorithm with an *E* value threshold of 10^−^^5^ (this value was applied in all subsequent BLAST analyses). For each of the nonredundant genes, the three best hits across the entire CS genome were extracted. A gene was deemed to represent a putative pericentric rearrangement if its three best hits were on opposite arms of the three chromosomes belonging to a given homoeologous group (Liu et al. 1992; Qi et al. 2006). For example, if the best three hits for a given gene were on chromosome arms 1AL, 1BL, and 1DS, the gene was judged to represent a putative transfer from 1DL to 1DS. For these genes, an additional seven hits were then considered, and the chromosomal locations were visually inspected again. Genetic map locations of wheat contigs were obtained from IWGSC (2014). It has been shown that a minute part of the centromeric region can be contained in both the long and short arms of ditelosomic lines for a given chromosome (Wicker et al. 2011). Therefore, genes detected on both arms of a chromosome were not considered in this study. The ortholog of *Bradi2g14130.1* from *Brachypodium* was one such gene. It detected homoeologous sequences on both the long and short arms of chromosome 1A and it was not possible, based on the available data, to determine the arm from which it originated.

### Configuration of Chromosome 4A

As a consequence of the well-known cyclic translocations involving chromosomes 4A, 5A, and 7B, the arm ratio of chromosome 4A has been reversed (Liu et al. 1992; Devos et al. 1995; Mickelson-Young et al. 1995; Nelson et al. 1995; Miftahudin et al. 2004). As a result, discussion of the various historical states of this chromosome can become difficult to understand. To alleviate this confusion, we used “original 4AS” and “original 4AL” to refer to the arms of the ancestral version of this chromosome and “modern 4AS” and “modern 4AL” to refer to the modern configuration arm of this chromosome.

### Identification of Arm Locations on Chromosome 3B

The CS shotgun sequence was obtained from chromosome arms flow-sorted from double ditelosomic lines (all chromosome arms except 3B, 7AS, 7AL, and 5BS), ditelosomic lines (7AS and 7AL), or an isochromosome (5BL). Chromosome 3B was flow-sorted in its entirety (IWGSC 2014). The centromere on CS chromosome 3B was located between positions 265 and 387 Mb (Choulet et al. 2014). This position was used in determining arm locations of genes on this chromosome.

### Validation of Gene Locations by Polymerase Chain Reaction Amplification

For validating the chromosome (arm) locations of genes identified from the above analysis, the euploid and selected nullisomic-tetrasomic (Sears 1954; Sears et al. 1966) and ditelosomic (Sears ER and Sears L 1978) lines of CS were analyzed. Genomic DNA was extracted from 20-day-old seedlings using the method of hexadecyltrimethylammonium bromide (Murray and Thompson 1980). Primers specific to a gene located on a specific chromosome arm involved in a putative intrachromosomal rearrangement were designed based on the alignment of sequences from the three homoeo-alleles. Polymerase chain reaction (PCR) amplification was performed in 10 μl reaction mixtures containing 50 ng of genomic DNA, 200 μM of each dNTP, 0.2 μM of each primer, and 0.5 U of Taq DNA polymerase. The cycling parameters were 94 °C for 5 min to predenature, which was followed by 35 cycles of 94 °C for 45 s, 40 s at the appropriate annealing temperature (ranging from 50 to 68 °C depending on the primers, see supplementary table S1, Supplementary Material online), 72 °C for 1 min, and a final extension at 72 °C for 10 min. Amplification products were separated on 1.5% agarose gels.

## Results

Using the highly stringent criteria set in this study, 551 genes identified sequences on homoeologous chromosomes but on a different arm in one of the three homoeologues, indicative of putative pericentric rearrangements present in either CS or in the aneuploid lines that were the source of the shotgun sequence (supplementary table S2, Supplementary Material online). Of these, 147 were single-copy genes which detected a single locus on each of the three homoeologous chromosomes. The other 404 genes detected a minimum of four loci with three being located on the three chromosomes of a given homoeologous group, and the remaining loci belonging to different homoeologous group(s). Deletion bin locations of ESTs were available for 70 of the 551 genes (supplementary table S2, Supplementary Material online).

**Fig. 1.—:**
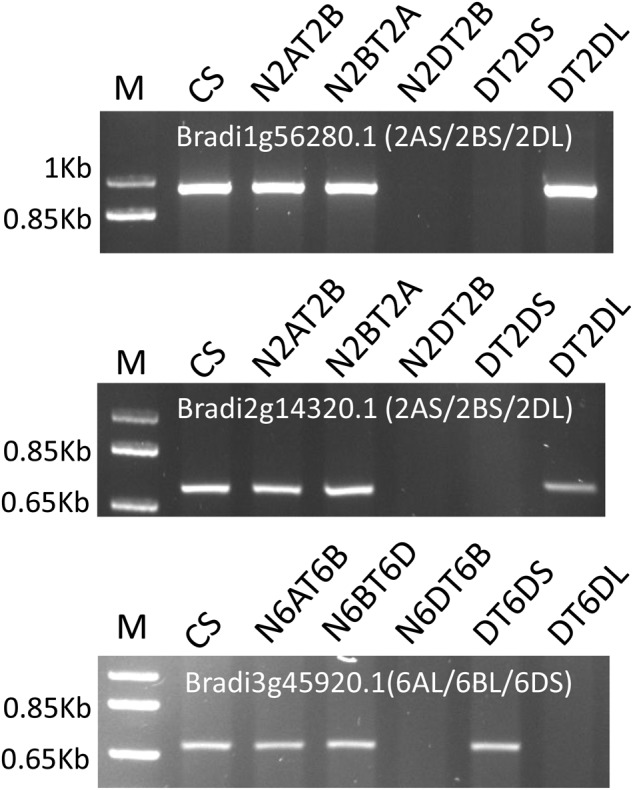
PCR profiles of the hexaploid wheat genotype CS nulli-tetrasomic (NT) and ditelosomic (DT) lines with primers for three of the genes involved in putative intrachromosomal rearrangements. Locations of the genes detected from the chromosome shotgun sequences are given in brackets following each gene. One kilobase pair (kb) plus DNA ladder was used as size marker (M).

Twenty of the genes indicative of putative intrachromosomal rearrangements were further assessed against the euploid, nullisomic-tetrasomic, and ditelosomic lines of CS (supplementary table S1, Supplementary Material online). Primers designed for four of these genes (*Contig66257*, *TRIUR3_10150*, *TRIUR3_35286, Bradi1g52600.1*) failed to amplify. PCR products were successfully obtained for the other 16 genes, and chromosome arm locations deduced from the chromosome shotgun sequences from the IWGSC were confirmed for all of them (fig. 1).

The 551 genes suggesting the existence of intrachromosomal rearrangements were located on 35 of the 42 chromosome arms of hexaploid wheat. Seven of these arms were represented by only a single gene each and the other 30 arms were represented by between 2 and 108 genes each (table 1 and supplementary table S2, Supplementary Material online). We considered that there was strong evidence for the presence of a pericentric inversion if 1) a minimum of six genes were transferred to the opposite arm of a given chromosome, 2) the genes were distributed over both arms with a minimum of three genes per arm, and 3) the inversion encompassed the centromere as demonstrated by the fact that at least some of the genes from both the short and long arms co-located or were tightly linked on the genetic map. Based on these criteria, 10 of the 21 CS chromosomes covering all homoeologous groups except group 5 were involved in pericentric rearrangements (table 1 and supplementary table S2, Supplementary Material online). A further four chromosomes, 4D, 5A, 6D, and 7D, carried 11 or more genes with nonstandard arm locations, the majority of which indicated the transfer of a segment of either the short arm to the long arm (4D and 5A) or of the long arm to the short arm (6D and 7D). Because there was no or limited evidence for the involvement of the other arm in the 4D, 5A, 6D, and 7D rearrangements, we refer to them as putative pericentric inversions.

**Table 1:** Number of Genes Involved in Intrachromosomal Rearrangements in Bread Wheat

Chr.^a^	Short to Long Arm	Long to Short Arm
**1A**	8	20
1B	3	3
1D	1	3
2A	2	1
**2B**	5	16
**2D**	15	3
3A	0	0
**3B****^b^**	55	94
3D	0	0
**4A**	108	25
**4B**	3	38
*4D*	20	0
*5A*	12	1
5B	1	1
5D	1	0
**6A**	6	15
**6B**	17	6
*6D*	1	13
**7A**	6	6
**7B**	24	7
*7D*	2	9
Total	551	

^a^Chromosomes in bold have pericentromeric inversions with strong support; Chromosomes in italics have putative pericentromeric inversions.

^b^Some of the nonstandard arm locations in 3B may not be caused by pericentric inversions (see text for details).

The existence of pericentric rearrangements in, potentially, all homoeologous groups prevents us from detailing each of them separately. We thus selected chromosome 1 as an example to show what information was drawn in supporting the claim of a given rearrangement. We also provide detailed descriptions of possible rearrangements in the group 4 chromosomes as the evidence supporting the presence of rearrangements in these chromosomes is less straightforward than for other chromosomes. For the remaining homoeologous groups, we only provide summary information. Full listings of the genes characterizing these rearrangements can be found in supplementary table S2, Supplementary Material online.

### Genes Indicating the Presence of a Possible Pericentric Rearrangement in Chromosome 1A

Arm locations of 28 genes indicated the presence of a pericentric inversion in chromosome 1A relative to 1B and 1D (supplementary table S2, Supplementary Material online). Twenty genes detected homoeologous sequences on 1AS/1BL/1DL (indicating transfers from 1AL to 1AS) and eight detected homoeologous sequences on 1AL/1BS/1DS (indicating transfers from 1AS to 1AL). Genetic map locations on chromosome 1A are known for at least 25 of these 28 genes (8 on 1AL and 17 on 1AS). Both short and long arm loci mapped to a single locus at 78.56 cM (supplementary table S2, Supplementary Material online), showing that these genes map to the pericentromeric region of chromosome 1A. Corresponding deletion bin-mapped ESTs were available for 3 of these 28 genes. The locations of BE604047 (deletion bins C-1BS10-0.5 and C-1DS3-0.48) and BF202643 (deletion bins C-1AS1-0.47, C-1BS10-0.50, and C-1DS3-0.48) in deletion bins adjacent to the centromere confirmed their pericentromeric location. However, the deletion bin data of BF202643 did not provide evidence of a pericentric rearrangement. Scrutiny of the autoradiographs from which the deletion bin locations were derived (http://wheat.pw.usda.gov/cgi-bin/westsql/map_locus.cgi, last accessed November 3, 2014) revealed that BF202643 was not hybridized to DNA of either the 1AS or 1AL ditelosomic line. Because a pericentromeric location in deletion bin mapping is concluded when a hybridizing fragment is present in all deletion lines, the deletion line data do not differentiate between a pericentromeric short arm or long arm location in the absence of chromosome arm information from ditelosomic lines. The 1AL-0.17–0.61 location of EST BE443622, which is also contradictory to the chromosome location obtained from the shotgun data, proved ambiguous upon scrutiny of the deletion line autoradiographs. Furthermore, the deletion bin-locations of BE443622 in the long arm (in deletion bin 1AL1-0.17–0.61) and a copy of BF202643 in the short arm (in deletion bin C-1AS1-0.47) are not supported by the available chromosome shotgun data which contained no sequences matching these ESTs in the arms defined by deletion bin mapping (supplementary table S2, Supplementary Material online), suggesting that the 1A deletion bin locations of these ESTs are incorrect.

### Genes Indicating the Presence of Pericentromeric Rearrangements in the Homoeologous Group 4 Chromosomes

In analyzing possible rearrangements for the homoeologous group 4 chromosomes, it is critical to remember that many genes in the modern short and long arms of chromosome 4A are homoeologous with those in the long and short arms, respectively, of chromosomes 4B and 4D due to a complex set of rearrangements involving chromosomes 4A, 5A, and 7B (Ma et al. 2013). Thus, expected arm locations for the majority of loci mapping to the group 4 chromosomes are 4AL/4BS/4DS and 4AS/4BL/4DL. We found five alternative patterns, including 4AS/4BS/4DS (25 genes), 4AL/4BS/4DL (20 genes), 4AL/4BL/4DL (108 genes), 4AS/4BS/4DL (38 genes), and 4AL/4BL/4DS (3 genes) (supplementary table S2, Supplementary Material online).

**Fig. 2.—:**
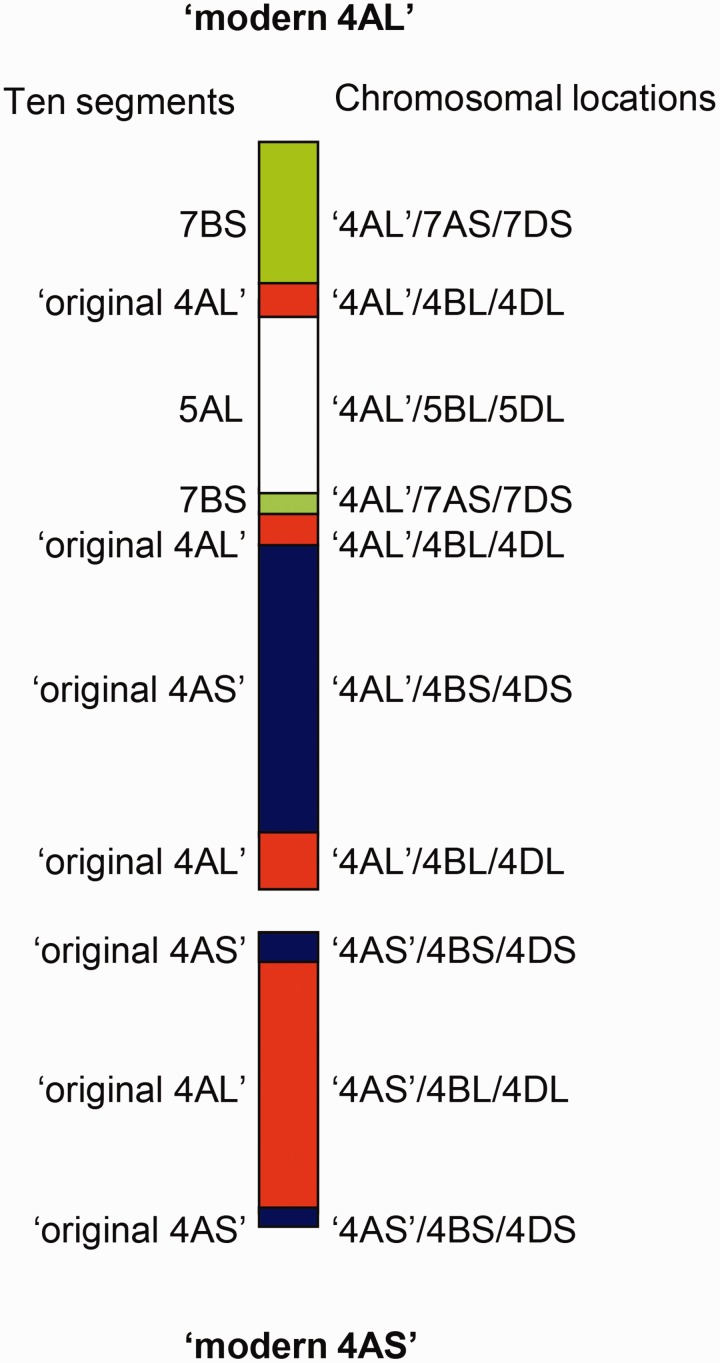
Configuration of the modern chromosome 4A which consists of ten segments. The chromosome arms from which the fragments originated are indicated to the left and matching sequences on homoeologous chromosome arms are indicated on the right.

Genes with locations on 4AS/4BS/4DS could either have been derived from a distal segment of the original 4AS that had not been involved in the complex 4AL/5AL/7BS translocation (the presence of such a fragment had been hypothesized but not yet been proven) (Ma et al. 2013) or represent the transfer of a segment from the modern 4AL to the modern 4AS (fig. 2). Map locations for most of the 25 4AS/4BS/4DS genes on chromosomes 4A and 4D are unknown. Their locations in wheat chromosome 4B, however, indicate that these genes represent at least two separate segments, one derived from the distal chromosome region providing the first evidence of the presence of an original 4AS fragment at the end of the modern 4AS (genes with map locations ranging from 2.86 to 4.98 cM on 4B) and the other from the centromeric region indicative of a rearrangement close to the centromere of chromosome 4A (genes with map locations ranging from 64.89 to 65.7 cM on 4B). Similarly, at least two chromosome segments, one in the centromeric region and one more distally located, were identified that have marker locations 4AL/4BL/4DL. The centromeric segment is characterized by nine genes that map to the region 64.89–65.7 cM on wheat chromosome 4B. These markers indicate the transfer of a segment of the modern 4AS to the modern 4AL. This rearrangement is supported by the location of ESTs BE637507 and BG274862, both of which map to deletion bins C-4AL-12-0.43, C-4BL1-0.71, and C-4DL9-0.31. The existence of a segment of the original 4AL on the modern 4AL arm is indicated by 99 genes with arm locations 4AL/4BL/4DL and genetic map locations in the region 74.63–94.55 cM on 4BL, several of which have supporting deletion bin mapping data (supplementary table S2, Supplementary Material online).

In addition to genes with locations 4AS/4BS/4DS and 4AL/4BL/4DL, 20, 38, and 3 genes were identified with homologues on 4AL/4BS/4DL, 4AS/4BS/4DL, and 4AL/4BL/4DS, respectively, that were derived from centromeric regions based on their genetic map location on chromosome 4B (at ∼65 cM) (supplementary table S2, Supplementary Material online). Although these genes mapped distally on the chromosome 4D linkage map (at ∼11.6 cM) (supplementary table S2, Supplementary Material online), they nevertheless were located in the centromeric region as indicated by the comapping of short and long arm loci. Where available, deletion map data confirmed that these genes were located in centromeric chromosome bins (supplementary table S2, Supplementary Material online). Of the two ESTs corresponding to genes with arm locations 4AL/4BS/4DL, one had been bin-mapped to C-4AL12-0.43 and the other to C-4DL9-0.31. They confirmed the 4AL and 4DL location obtained from the shotgun sequence data, but the deletion mapping provided no information on the presence of a potential pericentromeric rearrangement on 4D relative to 4A and 4B. Deletion mapping also confirmed the 4AS and 4DL locations of five genes identified from the shotgun sequence to have homologues on 4AS/4BS/4DL. However, there was some discrepancy in the 4B arm location for these ESTs. One EST, BE405597, had been mapped to 4BS, in agreement with the arm location from the shotgun sequence data, but two others (BE591915 and BE446061) had been allocated to chromosome arm 4BL. Similarly, deletion mapping of one EST confirmed the 4AL and 4DS locations of a gene identified in our study to have arm locations 4AL/4BL/4DS, but again there was a discrepancy in the 4B arm location between the deletion bin data and the shotgun sequence data. As for the group 1 data, scrutiny of the autoradiographs from which the deletion bin locations had been derived (http://wheat.pw.usda.gov/cgi-bin/westsql/map_locus.cgi, last accessed November 3, 2014) confirmed the arm locations on 4A and 4D of these ESTs, and the 4BS location for BE406512, but not the 4BL locations for ESTs BE591915 and BE446061 (ESTs with homologues in the shotgun data of chromosome arms 4AS, 4BS, and 4DL), nor the 4BS location for BE497635 (EST with homologues in the shotgun data of chromosome arms 4AL, 4BL, and 4DS).

### Rearrangements on Homoeologous Groups 2, 3, 5, 6, and 7

Based on our criteria for defining a pericentric inversion that at least six tightly linked genes, distributed over both arms with a minimum of three genes per arm, had nonstandard arm locations on homoeologous chromosomes, pericentric inversions were identified in chromosomes 2B, 2D, 3B, 6A, 6B, 7A, and 7B (supplementary table S2, Supplementary Material online). If we drop the second criterion, putative pericentromeric rearrangements may also have occurred in chromosomes 5A, 6D, and 7D. As for the group 1 and group 4 chromosomes, when there was a discrepancy between the chromosome arm locations obtained from the shotgun data and from the deletion line mapping, scrutiny of the available autoradiographs (http://wheat.pw.usda.gov/cgi-bin/westsql/map_locus.cgi, last accessed November 3, 2014) showed that the relevant ditelosomic lines were either missing or could not be scored unambiguously. Most likely, in these cases, arm locations had been allocated to homoeologous chromosomes based on expected arm relationships (e.g., if a gene mapped to 2AS and 2DS, a 2BS location was automatically assumed). The exceptions were one EST (BE314068) on the homoeologous group 2 chromosomes which had a confirmed deletion bin location on 2DS1-0.33–0.47 while the shotgun sequence indicated 2AS/2BS/2DL locations, and 11 ESTs on the homoeologous group 3 chromosomes, three with confirmed deletion bin locations on either C-3BS1-0.33 or 3BS1-0.33–0.57 that were allocated to the long arm of chromosome 3B and eight with confirmed deletion bin locations on C-3BL2-0.22, 3BS2-0.22-0.50, or 3BL7-0.63–1 that were allocated to the short arm of 3B in our study.

## Discussion

### Pericentromeric Inversions Are a Frequent Occurrence

Pericentromeric inversions had previously been identified on chromosomes 2B (based on 2 markers), 3B (4 markers), 5A (6 markers), and 6B (4 markers) (Conley et al. 2004; Miftahudin et al. 2004; Qi et al. 2006). Our data provide support for the inversions on chromosomes 2B, 5A, and 6B (supplementary table S2, Supplementary Material online) but the presence of the 3B inversion could not be determined. Although we identified a large number of markers in our study with arm locations 3AS/3BL/3DS (55 genes) and 3AL/3BS/3DL (94 genes), it seemed unlikely that these nonstandard arm locations were caused by a pericentromeric inversion. First, the genes with nonstandard arm locations were not limited to the centromeric region, but were spread over 106 cM on the 3B genetic map (supplementary table S2, Supplementary Material online). Furthermore, for all genes with nonstandard arm locations for which corresponding ESTs and deletion bin data were available, the 3B arm locations obtained from the sequence data and the deletion data disagreed. Scrutiny of the hybridization patterns of the ESTs on the 3B ditelosomic lines (http://wheat.pw.usda.gov/cgi-bin/westsql/map_locus.cgi, last accessed November 3, 2014) confirmed that the recorded arm locations were correct. In contrast to the other chromosomes where shotgun sequencing was conducted on individual chromosome arms derived from aneuploid lines, the shotgun sequence of chromosome 3B was obtained from a 3B chromosome flow-sorted from euploid CS. Consequently, the location of the centromere was unknown. However, a reference sequence had recently been assembled for chromosome 3B on which the centromere was placed in a 122 Mb region flanked by positions 265 and 387 Mb (Choulet et al. 2014). The 3B arm locations in our study were determined by locating the genes that were identified in the 3B shotgun sequence to the reference genome. Genes that were located in the region 1–265 Mb were allocated to 3BS and genes with locations in the region 387–774 Mb were allocated to 3BL. A possible explanation for the discrepancy in arm locations obtained from the sequence data and the deletion mapping data, and for the presence of genes with nonstandard arm locations across 3B is that some of the sequence scaffolds were incorrectly placed in the 3B pseudomolecule. We therefore did not further analyze chromosome 3B for potential rearrangements.

In addition to the previously identified 2B, 5A, and 6B inversions, our data provide strong evidence for at least seven other chromosomes and some evidence for a further three chromosomes having undergone pericentromeric rearrangements. A significant implication of these results is that, although much can be learned from the approach of comparative genomics in ordering and orientating sequence contigs (Berkman et al. 2012; Hernandez et al. 2012), such an approach will have only limited power in chromosomes that are structurally rearranged. An accurate sequence assembly of a given wheat chromosomes may have to come from the establishment of a physical map and sequencing of a minimum tiling path as was done for chromosome 3B (Paux et al. 2008). However, even then, ordering of sequence scaffolds remains a challenge as indicated by the likely misplacement of scaffolds in the 3B reference sequence. Extensive rearrangements will also complicate efforts of comparative genomics-based gene cloning especially in the pericentromeric regions.

### Are the Pericentric Inversions Present in Euploid CS?

Except for chromosome 3B, the shotgun sequence was obtained from individual chromosome arms flow-sorted from aneuploid, mostly double ditelosomic lines. Pericentric inversions identified in earlier studies were also based on observations made in aneuploid, mostly ditelosomic lines. So, an important question was whether these rearrangements characterized euploid CS or were specific to the aneuploids analyzed. Because double ditelosomic lines are generated by crossing long and short arm ditelosomic lines, the presence of the same rearrangement in both ditelosomic and double ditelosomic lines does not provide proof that this rearrangement was present in the euploid CS line that give rise to the aneuploids. Chromosome aberrations in the aneuploid CS stocks are not uncommon (Devos et al. 1999; Wicker et al. 2011). Considering that even T-DNA insertions can cause high rates of chromosomal translocations (Clark and Krysan 2010), breaking chromosomes during the production of ditelosomic lines likely had drastic effects on chromosome structures. Thus, caution should be exercised when extrapolating structural information from aneuploid stocks to euploid CS.

**Fig. 3.—:**
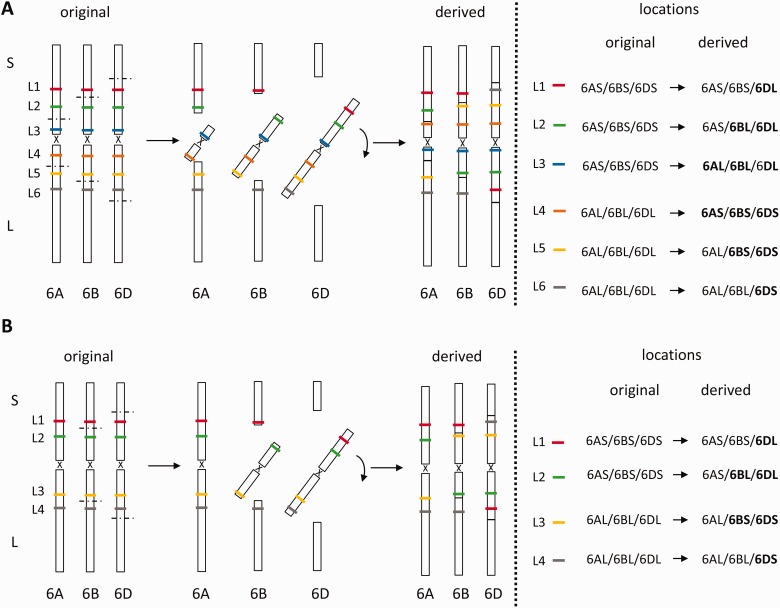
Possible nonstandard arm locations derived from pericentric inversions from a single genotype: (*A*) showing the four types of nonstandard arm locations (6AS/6BS/6DL, 6AS/6BL/6DL, 6AL/6BS/6DS, and 6AL/6BL/6DS) derived from pericentromeric inversions that occurred on chromosomes 6A, 6B, and 6D, with the size of the inverted segments on both the short and long arms being larger on 6D than on 6B than on 6A; and (*B*) showing that the same four types of nonstandard arm locations would be obtained if pericentromeric inversions occurred in two of the three.

Although there is no easy way to discern whether any single rearrangement is present in euploid CS, our data provide some indication that at least some of the rearrangements must have occurred in the aneuploids. When considering each chromosome independently, pericentric inversions appear to have occurred in only a single chromosome in the homoeologous group 1 and group 5 chromosomes, in two homoeologues in the group 2 chromosomes and in all three homoeologues in the group 4, 6, and 7 chromosomes. However, if pericentromeric inversions had taken place in all three homoeologous chromosomes in euploid CS, genes that were encompassed by all three inversions would appear as being nonrearranged, that is would be located on, for example, chromosome arms 6AS/6BS/6DS and 6AL/6BL/6DL or, in case of the group 4 chromosomes, on 4AL/4BS/4DS and 4AS/4BL/4DL. Consequently, the occurrence of pericentric rearrangements in all three chromosomes within a homoeologous group would yield only four patterns of nonstandard arm locations. For example, if pericentromeric inversions occurred on chromosomes 6A, 6B, and 6D, with the size of the inverted segments on both the short and long arms being larger on 6D than on 6B than on 6A, one would expect to find markers with the nonstandard arm locations 6AS/6BS/6DL, 6AS/6BL/6DL, 6AL/6BS/6DS, and 6AL/6BL/6DS (fig. 3*A*). The same nonstandard arm locations would be obtained if pericentromeric inversions occurred in two of the three homoeologs (i.e., in chromosomes 6B and 6D with the 6D inversion spanning a larger region than the inversion on 6B). Arm locations will vary depending on the chromosomes involved and on the relative size of the inverted segments (fig. 3*B*), but the maximum number of nonstandard arm locations that can be obtained from a single genotype is four. The fact that at least five patterns of nonstandard arm locations were identified on the group 4, 6, and 7 chromosomes strongly suggests that at least some of these rearrangements are not present in euploid CS but occurred during the generation of the ditelosomic or double ditelosomic lines.

### Inversion Breakpoints Are Limited to Pericentromeric Regions

The approach used in the current study can only detect rearrangements that encompass the centromere. Therefore, we cannot compare the relative frequency of paracentric and pericentromeric inversions. However, the large number of pericentromeric inversions identified allows us to examine the distribution of the inversion breakpoints along the chromosomes. Interestingly, the genetic map positions of the genes encompassed by the pericentromeric rearrangements showed that all inversion breakpoints were located within regions of very low recombination rates. Previous studies have shown that, relative to the distal regions, the proximal chromosome regions of wheat have greatly reduced recombination rates and significantly lower levels of DNA polymorphism (Dvořák et al. 1998, 2004; Choulet et al. 2014). Deletion bin-mapping also showed that the majority of EST-dense regions are in the distal parts of chromosomes and most of the agronomically important genes are located in the EST-dense regions (Qi et al. 2004; Rustenholz et al. 2011). The high repeat content of the low-recombinant pericentromeric regions may be one factor explaining why the inversion breakpoints are largely limited to those regions. Several studies have shown the involvement of inverted repeats in inversions (Carvalho et al. 2011; Mizuno et al. 2013), and such sequences would be expected to be present at much higher frequencies in repeat-rich centromeric and pericentromeric regions compared with the more distal gene-rich chromosomes regions. Selection may also play a role. Inversions limited to the gene-poor pericentromeric regions will encompass far fewer genes than inversions that extend into gene-rich distal chromosome regions. The latter type of inversions are more likely to affect the fitness of an organism (Gordon et al. 2007).

Only a small proportion of the genes representing putative rearrangements have matching ESTs that have been physically mapped using deletion bins, the locations of genes derived from deletion bin mapping and shotgun sequences do not always agree (supplementary table S2, Supplementary Material online), and the map resolution for the genes anchored on wheat chromosomes is still poor. Thus, reliable identification of genes immediately flanking the breakpoints to precisely characterize the majority of the putative rearrangements may have to wait.

### Gene Numbers Representing Intrachromosomal Rearrangements Were Likely Underestimated

Despite the fact that 551 genes with nonstandard chromosome arm locations were identified, there are at least four reasons why our study likely underestimated the number of genes encompassed by intrachromosomal rearrangements. First, as mentioned earlier, the identification of rearrangements was based on arm locations of genes on homoeologous chromosomes. This method can only identify genes representing pericentric rearrangements and lacks the power to reveal any paracentric changes. Second, only genes with the best three BLASTn hits on the three chromosomes belonging to a given homoeologous group were considered. This criterion prevented the inclusion of a large number of genes that detected homologues on all three homoeologous chromosomes that were not the best hits. For example, genes not considered included those which detected matching sequences, in order of decreasing sequence identity, on 1AL, 1BL, 3BS, and 1DS. However, identifying all genes involved in intrachromosomal rearrangements was not the main objective in this study. Furthermore, including only genes with top-three hits on homoeologous chromosomes increased the stringency of our analysis. Indeed, PCR-based validation of 16 sets of homoeologous arm locations showed all of them to be correct. Third, none of the genes having matching sequences on both arms for any of the chromosomes were included in this study for the reason that previous data had shown a limited number of genes in the centromeric regions to be present on both the long and short arm ditelosomic lines (Wicker et al. 2011). Most likely, when chromosome breakage occurs to give rise to telosomic lines, the breakpoint will be pericentromeric rather than centromeric as a functional centromere is needed for a telosome to be transmitted to the next generation. Although the breakpoints will almost certainly be different in each of the three homoeologues, this should not affect our results, in particular as genes with locations on both the short and long arm ditelosomic lines were excluded from our analysis. However, we cannot rule out that some of the genes with matching sequences on both arms arose from the duplication of individual genes or of chromosome segments. Fourth, genes also not considered in this study were those that detected homoeoloci in the shotgun sequence of only one or two of the three homoeologous chromosomes. Clearly, the available chromosome shotgun sequences are not completely covering each of the three hexaploid wheat subgenomes (IWGSC 2014).Thus, additional genes, possibly representing additional rearrangements, will be likely revealed in future studies.

### Genes Indicating the Presence of a Terminal Segment in the Modern 4AS Homoeologous to the Terminal Segments of 4BS and 4DS

The first indication that the terminal segment of the modern 4AS is homoeologous to the terminal segments of 4BS and 4DS came from a study of induced chromosome pairing. It was observed that in some pollen mother cells of 5B-deficient plants the modern 4AS was weakly associated with the termini of 4BS and 4DS, although the numbers of such cells were small (Naranjo et al. 1987). The locations of two deletion bin-mapped ESTs, BE518074, and BE494743, provided further support for this homoeologous relationship (Miftahudin et al. 2004). However, both of these ESTs detected matching sequences on multiple chromosomes in addition to those on the homoeologous group 4 chromosomes. Thus, conclusive evidence for the presence of a segment of the original 4AS on the modern 4AS following the occurrence of the otherwise well characterized chromosomal inversion on 4A (Naranjo et al. 1987; Anderson et al. 1992; Devos et al. 1995; Mickelson-Young et al. 1995; Nelson et al. 1995; Berkman et al. 2013; Ma et al. 2013) was still lacking (Liu et al. 1992; Ma et al. 2013).

Results from this study show unequivocally that the terminal segment of the modern 4AS is indeed homoeologous to the terminal segments of 4BS and 4DS. This terminal segment contains at least 15 genes. These genes do not only have the 4AS/4BS/4DS pattern of arm locations but, judging by the linkage map location of their counterparts on chromosome 4B, are all located terminally on these chromosome arms (supplementary table S2, Supplementary Material online). Thus, taking into account the chromosome 4A structure derived from previous studies (Ma et al. 2013), “modern 4A” should consist of at least ten segments from four different chromosome arms (fig. 2).

## Conclusions

Here, we report the likely existence of extensive pericentric rearrangements in the widely used reference genotype of hexaploid wheat, CS, revealed by detailed analysis of the CS chromosome arm shotgun sequences. Breakpoints for the majority of these pericentric inversions are located in the low-recombinant gene-poor pericentromeric regions. Because sequence assembly of shotgun sequence in repeat-rich regions such as wheat is largely limited to genic regions, ordering and orientating of sequence contigs is typically done using a comparative approach (chromosome zipper method). Structural differences among the three hexaploid wheat subgenomes will remain largely undetected using this approach, and consequently, chromosome zippers will likely have incorrect gene orders in rearranged regions. Results from our study indicate that at least some of the chromosomal rearrangements detected likely occurred during the production of the aneuploid stocks used for sequencing. Clarifying the possible differences in chromosomal structure between these aneuploid lines and the euploid is a must as sorting chromosome arms based on the use of ditelosomic lines has been the foundation of the current effort in sequencing the bread wheat genome.

## Supplementary Material

Supplementary tables S1 and S2 are available at *Genome Biology and Evolution* online (http://www.gbe.oxfordjournals.org/).

Supplementary Data
