# Supplementary material for: Extensive Pericentric Rearrangements in the Bread Wheat (Triticum aestivum L.) Genotype “Chinese Spring” Revealed from Chromosome Shotgun Sequence Data
Source: Genome Biol Evol. 2014 Oct 27;6(11):3039–48. doi: 10.1093/gbe/evu237 (PMC4255769; doi:10.1093/gbe/evu237)
Supplement: Supplementary Data [file supp_6_11_3039__index.html]

Extensive pericentric rearrangements in the bread wheat (Triticum aestivum L.) genotype ‘Chinese Spring’ revealed from chromosome shotgun sequence data — Extensive Pericentric Rearrangements in the Bread Wheat (Triticum aestivum L.) Genotype “Chinese Spring” Revealed from Chromosome Shotgun Sequence Data — Supplementary Data 

# Extensive Pericentric Rearrangements in the Bread Wheat (*Triticum aestivum* L.) Genotype “Chinese Spring” Revealed from Chromosome Shotgun Sequence Data

## Supplementary Data

files

**Files in this Data Supplement:**

- Supplementary Data - xlsx file
- Supplementary Data - xlsx file
